# High-power graphene mode-locked Tm/Ho co-doped fiber laser with evanescent field interaction

**DOI:** 10.1038/srep16624

**Published:** 2015-11-16

**Authors:** Xiaohui Li, Xuechao Yu, Zhipei Sun, Zhiyu Yan, Biao Sun, Yuanbing Cheng, Xia Yu, Ying Zhang, Qi Jie Wang

**Affiliations:** 1 Center for OptoElectronics and Biophotonics, School of Electrical and Electronic Engineering & The Photonics Institute, Nanyang Technological University, 50 Nanyang Ave., 639798, Singapore; 2 Department of Micro- and Nanosciences, Aalto University, PO Box 13500, FI-00076 Aalto, Finland; 3 Singapore Institute of Manufacturing Technology, 71 Nanyang Drive, 638075 Singapore; 4 School of Physics and Information Technology, Shaanxi Normal University, Xi’an 710062, P.R. China

## Abstract

Mid-infrared ultrafast fiber lasers are valuable for various applications, including chemical and biomedical sensing, material processing and military applications. Here, we report all-fiber high-power graphene mode-locked Tm/Ho co-doped fiber laser at long wavelength with evanescent field interaction. Ultrafast pulses up to 7.8 MHz are generated at a center wavelength of 1879.4 nm, with a pulse width of 4.7 ps. A graphene absorber integrated with a side-polished fiber can increase the damage threshold significantly. Harmonics mode-locking can be obtained till to the 21^th^ harmonics at a pump power of above 500 mW. By using one stage amplifier in the anomalous dispersion regime, the laser can be amplified up to 450 mW and the narrowest pulse duration of 1.4 ps can be obtained simultaneously. Our work paves the way to graphene Tm/Ho co-doped mode-locked all-fiber master oscillator power amplifiers as potentially efficient and economic laser sources for high-power laser applications, such as special material processing and nonlinear optical studies.

Long-wavelength (>1.55 μm) ultrafast lasers can potentially be applied in various applications, such as free-space optical communication due to atmospheric transparency window, molecular spectroscopy, medical diagnostics, laser surgery, long-range radar, and gas detection^1^. They can also be applied in high-efficient industrial material processing of various special materials, such as plastics, glasses, and leather because of their strong absorption in the mid-infrared range^2^,^3^,^4^. Compared to the conventional solid-state laser counterpart, fiber laser recently attracts considerable interests due to its alignment-free structure, compactness, efficient heat dissipation, and superior output performance (such as high beam quality, environmental stability, and flexible output).

Mode locking is the most typical approach to generate ultrashort pulses. Various methods have been reported to achieve mode locking: Nonlinear polarization rotation (NPR) is the one of the most commonly used methods for fiber laser mode locking, which have been studied for many years^5^. Other methods, such as nonlinear amplifying loop mirror (NALM), nonlinear optical loop mirror (NOLM) etc. have also been utilized to achieve mode locking^6^,^7^,^8^. Different materials, with ultrafast response time, have also been studied to achieve mode locking. Semiconductor saturable absorber mirror (SESAM) has been used as a type of saturable absorber (SA) for quite a long time^9^,^10^. However, it has some drawbacks, such as complex fabrication and packaging, low damaged threshold, high cost. Recently, single-wall carbon nanotubes (SWNT) and graphene^11^,^12^,^13^,^14^,^15^,^16^, have also been demonstrated as promising SAs for ultrafast pulse generation^17^,^18^,^19^. In particular, graphene has shown excellent performance to generate ultrashort pulses, such as broadband operation^20^,^21^, ultrafast recovery time^12^, ease of fabrication, and integration into all-fiber structure^19^,^22^. Diverse fabrication methods have also been utilized for graphene saturable absorber fabrication, such as micromechanical cleavage^23^, liquid phase exfoliation (LPE)^24^, chemical-vapor-deposition (CVD)^25^,^26^, and carbon segregation from silicon carbide^27^,^28^. Various integration methods have been demonstrated for graphene-based all-fiber device integration: such as directly sandwiched between two-fiber ends^29^, sprayed on the glass^30^, or the side polished fibers^31^.

In order to generate ultrashort pulse at wavelength above 1.55 μm and even up to 3.9 μm^32^,^33^, erbium (Er), thulium (Tm), holmium (Ho), or Tm/Ho-co-doped active fibers are utilized to offer appropriate gain region for fiber laser applications. Very recently, graphene ultrafast fiber lasers have been demonstrated at the mid-infrared spectral range. Tm-doped mode-locked fiber lasers based on graphene or multilayer graphene-polymer composite^34^,^35^,^36^,^37^,^38^, graphene oxide^31^, have been demonstrated. High pulse energy Tm/Ho-co-doped fiber laser has also been demonstrated^39^. However, most of the power level is limited to several mW levels and the peak power is less than hundreds of Watt, limiting their applications.

In this paper, we utilize the side-polished fiber together with graphene grown by CVD method to achieve mode locking in a Tm/Ho co-doped ring fiber laser. It is demonstrated that mode locking can be obtained at a center wavelength of 1879.4 nm, spectral bandwidth of 1.8 nm, and pulse width of about 4.7 ps. High-order harmonic mode locking (up to 164.6 MHz) can be obtained with the increase of pump power. After we use a Tm-doped fiber amplifier (TDFA) to boost the output power, the maximum output power is ~450 mW, corresponding to the peak power of about 9.7 kW. Our results shows graphene all-fiber Tm/Ho co-doped mode-locked fiber laser with single mode output and high peak power. These unique properties enable the advantages of compactness, good beam quality and high power output, which make them potential candidates in the molecular spectroscopy, nonlinear frequency conversion, and laser surgery applications.

## Results

**Figure 1:**
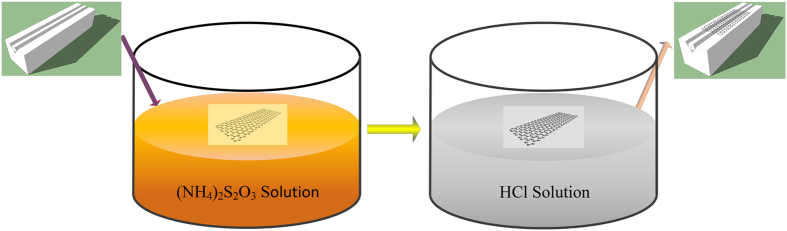
Transferred method for preparing graphene SA. The left part is schematics of the side-polished fiber without graphene. The MCVD grown graphene is dissolved in an aqueous (NH_4_)_2_S_2_O_3_ solution. The cleaned PMMA/graphene film is dissolved in 0.5 M HCl solution. The side-polished fiber is put at the bottom of PMMA/graphene film in the breaker to pick up the target. The right part is the side polished fiber with graphene.

CVD grown single layer graphene (SLG) (~20 × 20 mm^2^, Graphene Supermarket) is transferred onto a side-polished fiber embedded in a glass plate by a modified transfer process. Figure 1 shows the schematic of the transferred processes. As-fabricated SLG on the copper is spin-coated with poly (methyl methacrylate) (PMMA, average M_W_~996000 by GPC, Sigma Aldrich, dissolved in chlorobenzene with a concentration of 50 mg/mL), which is then cured at 110 °C for 10 min. The underneath Cu foil is then dissolved in an aqueous (NH_4_)_2_S_2_O_3_ solution (10%). The remaining PMMA/graphene film is cleaned by repeatedly dipping it in deionized water, and then a 0.5 M HCl bath for 5–10 min. Then, the PMMA/graphene film is picked up through the target side-polished fiber. After drying in the ambient condition, some PMMA solution is dropped onto the side-polished fiber to dissolve the pre-coated PMMA and flatten out winkles on the graphene. The PMMA is then dissolved in acetone, leaving SLG on glass with side polished fiber.

**Figure 2:**
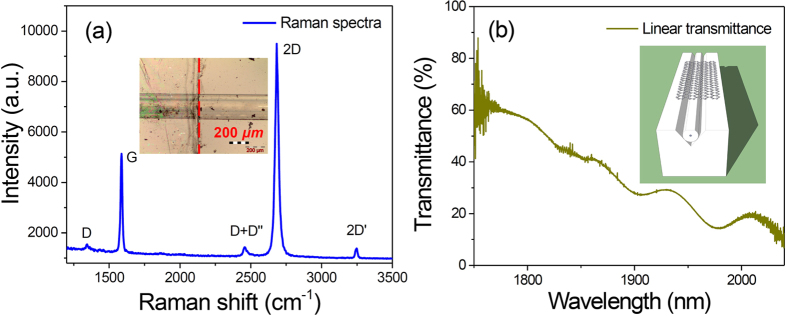
(**a**) The Raman spectrum of the CVD grown graphene SA device. Inset: the microscope image of our transferred graphene on the side polished fiber with the scalar bar of 200 μm. (**b**) Linear transmittance of our fiber-integrated graphene saturable absorber. Inset: schematic diagram of a side-polished fiber covered with graphene as a SA device.

After transfer, the graphene on the side-polished fiber was characterized by Raman spectroscopy (under 532 nm excitation) and absorption microscopy. A representative Raman spectrum of the transferred graphene on side-polished fiber is shown in Fig. 2(a). 2D peak has a single Lorentzian shape. The ratio of G and 2D Raman peaks (I_G_/I_2D_) is 0.54, which is recognized as intrinsic single layer graphene^40^. The negligible D, D+D″ and 2D′ band indicates high quality of CVD SLG^41^. The inset shows the microscope image of our transferred graphene on side-polished fiber. The left part of the red-dashed line in the image is with SLG which covers the side-polished fiber. The right part of red-dashed line is without SLG. In addition, we use a home-made broadband Amplified spontaneous emission (ASE) source (from 1700 to 2100 nm) to measure the linear transmittance of the side polished fiber integrated with SLG. The result is shown in Fig. 2(b). The transmittance at 1880 nm is about 40%. The inset of Fig. 2(b) shows the schematic diagram of side-polished fiber covered with SLG SA.

**Figure 3:**
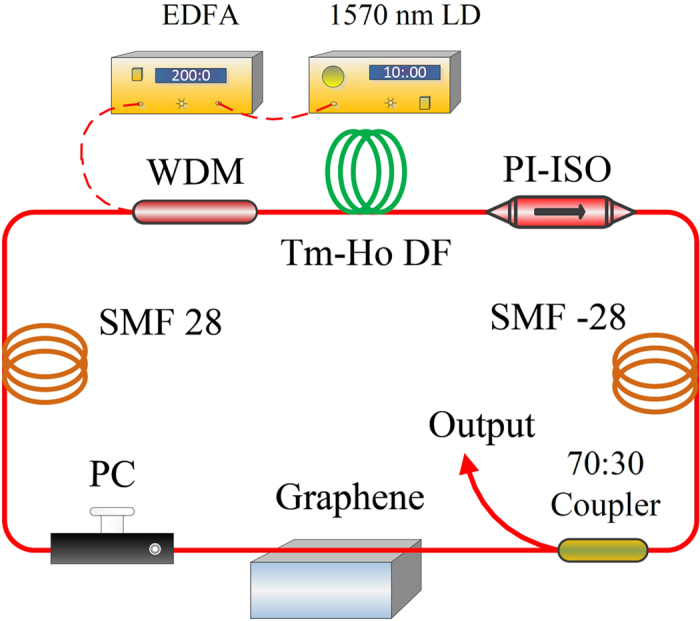
The experimental setup of the Tm/Ho co-doped fiber laser based on side-polished fiber with graphene .

Figure 3 shows the schematic of the proposed fiber laser based on our fiber-integrated graphene SA. The fiber laser is composed of several fiber components, including a 1.5-m-long Tm/Ho co-doped fiber (from CorActive company) with absorption of 137 dB/m at 790 nm, a 1570/1950 wavelength division multiplexing (WDM) coupler, a polarization insensitive isolator (PI-ISO), one polarization controller (PC), a 70:30 optical coupler (OC), and a side-polished fiber covered with SLG. A 1570-nm laser diode with ~10-mW output power amplified with C+L band erbium-doped fiber amplifier (EDFA), which has the capability of amplifying the signal to more than 1 W, is used as pumping source for the laser system. The pulse train is exported from the fiber cavity through 30% port of OC. All the fiber components inside the laser system are fusion spliced, with a total length of about 26.2 m.

When the pump power reaches 371 mW, mode-locking can be self-started. When we decrease the pump power, pulse train can be maintained till 326 mW, which is due to the hysteresis effect^42^. The hysteresis effect in the experiment is identical to other rare earth-doped passively mode-locked fiber lasers, such as Yb-doped fiber laser in the 1-μm region, and Er-doped fiber laser in the 1.5-μm region.

**Figure 4:**
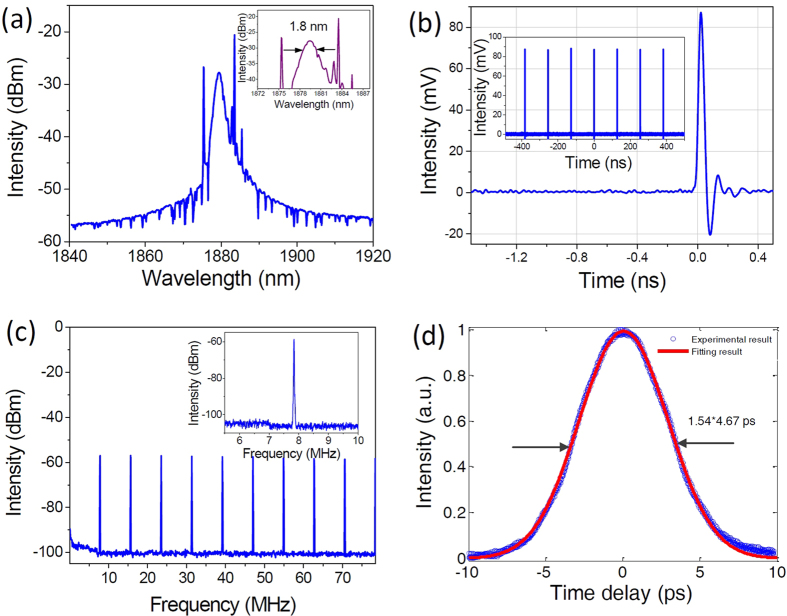
The output characteristics of Tm/Ho co-doped mode-locked fiber laser at the fundamental repetition rate. (**a**) Output spectrum, inset: the spectral width is about 1.8 nm, (**b**) single pulse as observed from the high-speed oscilloscope, inset: corresponding pulse train, (**c**) RF spectra, inset: RF spectra at the fundamental frequency of ~7.8 MHz, (**d**) autocorrelation trace, with pulse width of 4.67 ps.

The typical output of the fiber laser (optical spectrum, oscilloscope trace, RF spectrum, and the corresponding autocorrelation trace) is shown in Fig. 4. Figure 4(a) shows the typical spectrum of fiber lasers with a center wavelength of 1879.4 nm. The spontaneous emission hump of single mode Tm/Ho co-doped fiber is located at 1880 nm when the length of the fiber is around 2 m, and thus the maximum gain can be obtained around this wavelength regime. Spectral sideband is observed, indicating that our fiber laser operates in the conventional soliton mode-locking regime. With the increase of the length of Tm/Ho co-doped fiber, the center wavelength can shift to longer wavelength side, which is due to secondary pumping phenomena in an un-pumped active fiber^43^. The inset of Fig. 4(a) shows the zoom-in spectrum, indicating spectral width of 1.8 nm. Figure 4(b) shows the oscilloscope trace of a single pulse obtained from a high-speed oscilloscope. The inset shows the pulse train with a period of 127.6 ns. Figure 4(c) shows the RF spectra with a span of 78 MHz. Nine frequency peaks can be observed within the range. The inset is the fundamental frequency of ~7.8 MHz. Figure 4(d) indicates that the pulse temporal profile is well fitted by a sech^2^ profile with a pulse width of 4.67 ps. The corresponding 3-dB spectral width is 1.8 nm, corresponding to a time-bandwidth product (TBP) of about 0.714. The TBP is larger than transform-limited sech^2^ pulse, which indicates the pulses are chirped. Compared with Ref. 31the output of our proposed fiber laser, with a total dispersion of about −1.757 ps^2^, is comparable. The pulse width can be further reduced by controlling the dispersion of the cavity.

**Figure 5:**
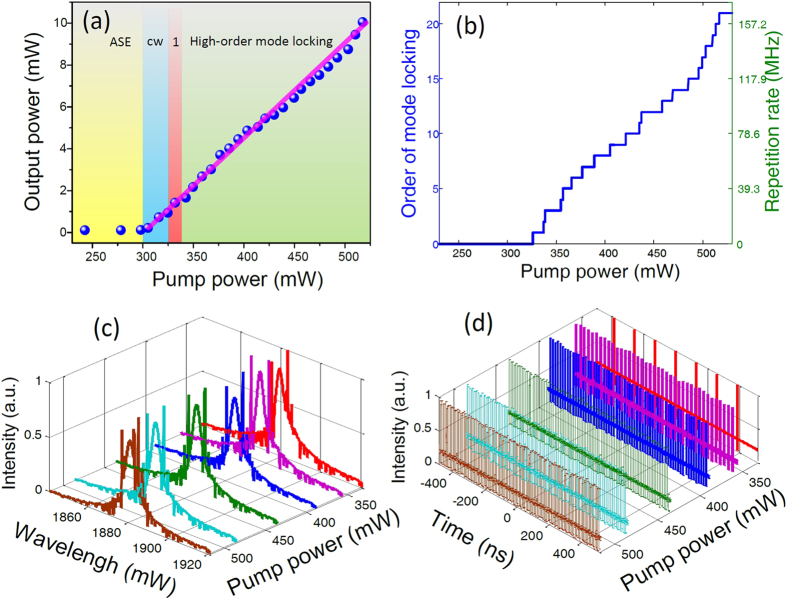
(**a**) The output power versus the pump power. Different color represents different operating regimes. (**b**) The repetition rate and orders of mode locking versus the pump power. The fundamental repetition rate to 21^th^ harmonics mode-locking evolution versus the pump power, (**c**) the spectral evolution, (**d**) corresponding pule train evolution.

With further increasing the pump power, our fiber laser can operate from the fundamental mode locking regime to the high-order harmonic mode-locking regime. Figure 5(a) shows the output power versus the pump power of our Tm/Ho co-doped fiber laser. The slope efficiency is around 4.8% through linear fitting as show in pink line. Considering the linear loss of around 60% induced by the side-polished fiber with SLG and other components, the slope efficiency is reasonable. Different operation regimes can be achieved under different pump power and are represented by using different colors. ASE regime is from 230 mW to 300 mW represented in the yellow color area. CW and unstable regimes are from 300 mW to 326 mW in blue color area. Fundamental mode locking regime can be obtained from 326 mW to 338.9 mW represented in the red color area. Number “1” is used to represent the fundamental mode locking regimes. High-order mode locking is in above 338.9 mW in green color. Figure 5(b) shows the repetition rate and orders of harmonic mode locking versus pump powers. Through increasing the pump power, the order of the mode locking increases. When the pump power is increased to 517.4 mW, our fiber laser can operate at the 21^th^ harmonics mode locking, which gives an output of 10.1 mW with a repetition rate of 164.6 MHz. Because of the limitation of the damage threshold of the fiber components (500 mW), the pump power are not further increased. Figure 5(c) shows the spectra evolution at the fundamental, 5^th^, 8^th^, 11^th^, 15^th^, and 21^th^ harmonic mode locking versus the different pump power. Figure 5(d) shows the corresponding pules-train evolution. Harmonics mode locking state can be partially explained by the soliton energy quantization theory^44^. Due to the soliton energy relocation and the interaction between the multiples solitons per round cavity round trip, the multiples at high pump power will form a special state, which have the equal separation in the fiber cavities, i.e. harmonics mode locking.

The absorber structure in our work is quite different from those with the sandwich structure. In our work, higher-order harmonic mode locking is observed till to the 21^th^ harmonic, which is limited by the damaged threshold of the WDM coupler rather than the absorber. The absorber with a sandwich structure, however, cannot bear such high pump power. Thus, it is difficult to observe higher-order harmonics mode locking based on the sandwich-structure absorber.

**Figure 6:**
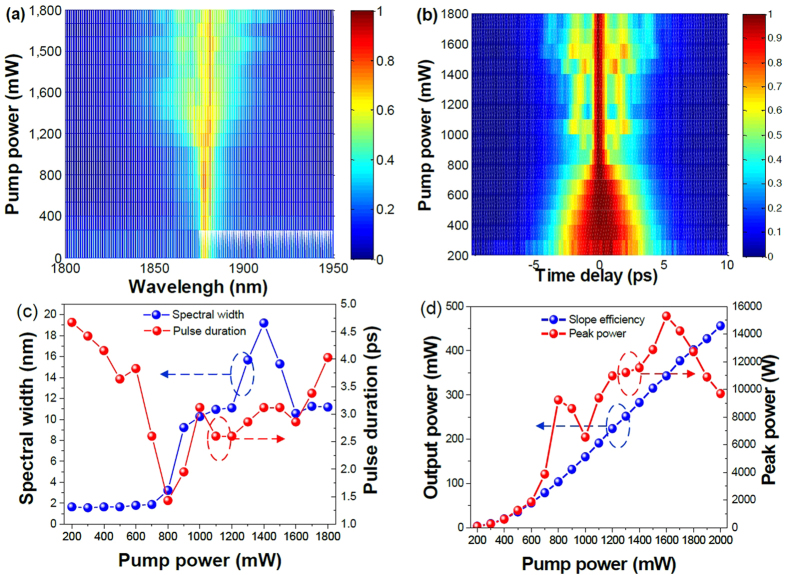
(**a**) The spectral evolution, (**b**) corresponding pulse evolution, (**c**) spectral width and pulse duration, (**d**) the output power and peak power versus the pump strength.

To increase the output power of our graphene based fiber laser, we use a home-made TDFA to amplify the seed laser. The maximum output power after amplification is ~450 mW by removing the residual pump power, when the passively mode-locked fiber oscillator operates at the fundamental mode locking (output power of the oscillator is 3 mW). The amplification coefficient is more than 21 dB. The spectral evolution at different output power is shown in Fig. 6(a). The spectra becomes broadening with the increase of the pump power. When the pump power is larger than 800 mW, the spectra are split and additional frequency components will generate as seen from the spectra. With further increasing the pump power of the TDFA, the spectra are broadened. More pair of humps will be generated in the frequency domain, which is due to the high nonlinear effect (such as modulation instability, self-phase modulation effect, etc). Simultaneously, the pulse will experience compressed processing, and multiple pulses process. Figure 6(b) shows the pulse evolution with the increase of the pump power. The pulse is compressed when the pump power increases. When the pump power is above 800 mW, the pulses become high order soliton pulses. More additional pulses can be generated with the increase of pump power. Figure 6(c) summarizes the spectral width and pulse-width evolution versus the pump power. We can see that below 800-mW pump power, the pulse becomes narrow and the spectra become broaden. Due to high-order nonlinear effect, the pulse is split into multiple pulses. With further increasing the pump power, more side humps are generated in the spectrum and additional sub-pulses are generated beside the main large pulse. Figure 6(d) shows the output power and the peak power versus the pump power of TDFA. We can see that the peak power becomes large with the increase of pump power. When the pump power is 800 mW, the peak power and average output power are 9.23 kW, 103.5 mW, respectively. By further increasing the pump power, high order soliton effect will make the pulse split into a main pulse and more sub pulses. In order to calculate the average peak power of the high-order soliton pulse, we assume the pulse width as the envelope width. The envelope width becomes wider because of high order-soliton pulse effect. When the pump power increases to 2 W, the output power is 456 mW. The peak power is estimated to be around 9.7 kW.

Conventionally, chirped pulse amplification (CPA) technique can overcome the pulse break-up, which is widely used in the normal-dispersion regime. After being amplified, the pulse can be further compressed due to the linear chirp of the pulse. Different from the CPA technique, the pulse can be amplified and compressed simultaneously in the anomalous dispersion regime. This can help to get shorter pulse than the transform-limited pulse due to the nonlinearity and high order soliton effect. Our approach provides a scheme to further compress the transform-limited pulse.

In order to demonstrate that the mode locking is solely mode locked by the SLG SA with graphene, we remove the side polished fiber with graphene or just remove the graphene on the side-polished fiber. No clear mode-locked pulse train or typical mode-locked spectrum can be observed. Only the CW regime was obtained from the laser cavity, which confirms that the mode locking is solely contributed by the side-polished fiber with graphene. The stability of our laser has been tested for a few days, no performance degradation was observed.

In conclusion, we demonstrate passively mode-locked high power Tm/Ho Co-doped fiber laser based on a side-polished fiber incorporated with CVD grown graphene. The obtained pulse width is about 4.7 ps, the spectral width is about 1.8 nm with Kerr side band, indicating that the fiber laser operates in the conventional soliton regimes. With further increase of the pump power, the Tm/Ho co-doped mode-locked fiber laser can operate in high-order harmonic mode locked states till to the 21^th^ order, which is limited by the damaged threshold of fiber components. The output power of the proposed fiber laser can be further amplified to more than 450 mW, corresponding to a peak power of about 9.7 kW. The high peak-power Tm/Ho co-doped mode-locked fiber laser, with single mode output and high beam quality, can be potentially applied with further development in applications such as laser surgery, medical diagnostics, and gas detection, etc.

## Method

### Measurement method

Output spectra of our fiber lasers are measured through an optical spectrum analyzer (OSA, Yokogawa AQ6375). Mode-locked pulse train and radio frequency (RF) spectra are detected by using a 33-GHz mixed signal oscilloscope (MSO, Tektronix, 73304DX), one signal source analyzer (SSA, Rohde & Schwarz FSUP26) coupled with 12-GHz photodetector (Newport 818-BB-51F), respectively. A wide-range autocorrelator (From Femtochrome FR-103HP) is used to detect output pulse width.

## Additional Information

**How to cite this article**: Li, X. *et al.* High-power graphene mode-locked Tm/Ho co-doped fiber laser with evanescent field interaction. *Sci. Rep.* **5**, 16624; doi: 10.1038/srep16624 (2015).
